# Metagenomic Discovery of “*Candidatus* Parvarchaeales”-Related Lineages Sheds Light on Adaptation and Diversification from Neutral-Thermal to Acidic-Mesothermal Environments

**DOI:** 10.1128/msystems.01252-22

**Published:** 2023-03-21

**Authors:** Yang-Zhi Rao, Yu-Xian Li, Ze-Wei Li, Yan-Ni Qu, Yan-Ling Qi, Jian-Yu Jiao, Wen-Sheng Shu, Zheng-Shuang Hua, Wen-Jun Li

**Affiliations:** a State Key Laboratory of Biocontrol, School of Life Sciences, Sun Yat-Sen University, Guangzhou, People’s Republic of China; b Guangdong Provincial Key Laboratory of Plant Resources, School of Life Sciences, Sun Yat-Sen University, Guangzhou, People’s Republic of China; c Southern Marine Science and Engineering Guangdong Laboratory (Zhuhai), School of Life Sciences, Sun Yat-Sen University, Guangzhou, People’s Republic of China; d Chinese Academy of Sciences Key Laboratory of Urban Pollutant Conversion, Department of Environmental Science and Engineering, University of Science and Technology of China, Hefei, People’s Republic of China; e School of Life Sciences, South China Normal University, Guangzhou, People’s Republic of China; f State Key Laboratory of Desert and Oasis Ecology, Xinjiang Institute of Ecology and Geography, Chinese Academy of Sciences, Urumqi, People’s Republic of China; California State University, Northridge

**Keywords:** archaea, DPANN, thermophily, ancestral traits

## Abstract

“*Candidatus* Parvarchaeales” microbes, representing a DPANN archaeal group with limited metabolic potential and reliance on hosts for their growth, were initially found in acid mine drainage (AMD). Due to the lack of representatives, however, their ecological roles and adaptation to extreme habitats such as AMD as well as how they diverge across the lineage remain largely unexplored. By applying genome-resolved metagenomics, 28 Parvarchaeales-associated metagenome-assembled genomes (MAGs) representing two orders and five genera were recovered. Among them, we identified three new genera and proposed the names “*Candidatus* Jingweiarchaeum,” “*Candidatus* Haiyanarchaeum,” and “*Candidatus* Rehaiarchaeum,” with the former two belonging to a new order, “*Candidatus* Jingweiarchaeales.” Further analyses of the metabolic potentials revealed substantial niche differentiation between Jingweiarchaeales and Parvarchaeales. Jingweiarchaeales may rely on fermentation, salvage pathways, partial glycolysis, and the pentose phosphate pathway (PPP) for energy conservation reservation, while the metabolic potentials of Parvarchaeales might be more versatile. Comparative genomic analyses suggested that Jingweiarchaeales favor habitats with higher temperatures and that Parvarchaeales are better adapted to acidic environments. We further revealed that the thermal adaptation of these lineages, especially Haiyanarchaeum, might rely on genomic features such as the usage of specific amino acids, genome streamlining, and hyperthermophile featured genes such as *rgy*. Notably, the adaptation of Parvarchaeales to acidic environments was possibly driven by horizontal gene transfer (HGT). The reconstruction of ancestral states demonstrated that both may have originated from thermal and neutral environments and later spread to mesothermal and acidic environments. These evolutionary processes may also be accompanied by adaptation to oxygen-rich environments via HGT.

**IMPORTANCE** “*Candidatus* Parvarchaeales” microbes may represent a lineage uniquely distributed in extreme environments such as AMD and hot springs. However, little is known about the strategies and processes of how they adapted to these extreme environments. By the discovery of potential new order-level lineages, “*Ca.* Jingweiarchaeales,” and in-depth comparative genomic analysis, we unveiled the functional differentiation of these lineages. Furthermore, we show that the adaptation of these lineages to high-temperature and acidic environments was driven by different strategies, with the former relying more on genomic characteristics such as genome streamlining and amino acid compositions and the latter relying more on the acquisition of genes associated with acid tolerance. Finally, by the reconstruction of the ancestral states of the optimal growth temperature (OGT) and isoelectric point (pI), we showed the potential evolutionary process of Parvarchaeales-related lineages with regard to the shift from the high-temperature environment of their common ancestors to low-temperature (potentially acidic) environments.

## INTRODUCTION

Archaea were among the earliest-emerging lineages of the tree of life (1, 2). Diversely found in all sorts of environments, archaea often predominate in extreme environments such as hot springs, acid mine drainage (AMD), and hypersaline environments (3). As a major archaeal lineage, DPANN archaea have uniquely small genomes with limited metabolic potentials and are thus considered to have a symbiotic lifestyle (4–12). However, albeit with metabolic deficiencies, they have been found to be ubiquitous in nature and are distributed mostly in oxygen-limited environments (5, 6, 13). These studies have revealed their unique and unneglectable roles in biogeochemical cycles (14) as well as mysterious interactions with their potential hosts.

“*Candidatus* Micrarchaeota” (Micrarchaeota here) and “*Candidatus* Parvarchaeota” (reassigned to the order “*Candidatus* Parvarchaeales” of the phylum “*Nanoarchaeota*” in GTDB r207 [https://gtdb.ecogenomic.org]) were first found in AMD, and the name archaeal Richmond Mine acidophilic nanoorganisms (ARMAN) was proposed due to their ultrasmall cells and genomes (15, 16). Phylogenetic inference in a later study showed that “*Ca.* Parvarchaeota” differs from “*Ca.* Micrarchaeota” as a separate lineage (9). Similar to Micrarchaeota, the metabolic potential of Parvarchaeota is versatile, with the possession of a diversity of capacities, such as glycolysis, the tricarboxylic acid (TCA) cycle, the electron transfer chain (ETC), and numerous polysaccharide degradation pathways, which were relatively less frequently seen in other DPANN archaea (9, 13). The presence of the ETC and the TCA cycle in oxygen-limited environments is possibly associated with the consumption of oxygen rather than aerobic respiration. Although initially discovered in AMD, other ARMAN archaea, Micrarchaeota, have tremendously expanded in phylogenetic diversity and have been found to exist in a variety of environments, including extreme habitats such as hot springs and radioactive sites as well as nonextreme habitats like underground water (17–19). In contrast, Parvarchaeota appear to inhabit a narrower range of habitats. Thus far, their genomes have been obtained exclusively from acidic and/or thermal environments (9, 20). However, their mechanisms of adaptation to harsh conditions and how they evolved to give rise to such metabolic versatility are still unclear.

Many archaeal lineages were considered to originate from high-temperature (thermal) environments. It is well supported that the early environment of the Earth was found to be of high temperature and pressure, where only hyperthermophiles (often seen in deep-rooted archaea and bacteria) would have survived under such circumstances (21). Growth condition characterizations of microorganisms are essential for understanding their physiology. However, basic growth conditions such as the optimal growth temperature (OGT) and optimal pH are inaccessible for archaea and bacteria without cultured representatives. Pieces of evidence also showed that the OGTs of bacteria and archaea can be well explained by the GC contents of rRNAs and amino acid compositions (22–24). Consequently, efforts to use genomic composition to reflect environmental conditions have been proposed as “reverse-ecology” approaches (25). In this sense, characteristics such as OGT and pH have been estimated by various methods. For example, the overrepresentation of 7 amino acids, Ile, Val, Tyr, Trp, Arg, Glu, and Leu (IVYWREL), was shown to be positively correlated with the OGTs of *Archaea* and *Bacteria* and subsequently could be used for OGT predictions (23). Other methods were based on amino acid compositions or a combination of 16S rRNA genes, tRNAs, open reading frames (ORFs), and protein sequences (26, 27). Further ancestral-state reconstructions have revealed that many archaeal ancestors adapt to high-temperature environments with high OGTs (28–30).

As one of the major deep-rooted archaeal lineages, the DPANN superphylum was shown to have a mesophilic origin in previous studies (30–32). However, this could be the result of the lack of representation of genomes in high-temperature environments. Thus, it is still unclear whether DPANN as a whole or at least some specific lineages like Parvarchaeota originated from high-temperature environments. In addition, if Parvarchaeota have a distribution uniquely in acidic habitats, whether it was a legacy of early colonization in acidic hot springs or a later adaptation after the dispersal to acidic habitats remains unknown.

Here, we reconstructed 28 metagenome-assembled genomes (MAGs) from hot spring samples from Tengchong, Yunnan, China. Using genome-resolved metagenomics analyses and applying comprehensive phylogenetic analyses, we identified 10 MAGs branched deeply within the Parvarchaeales, 4 MAGs that belong to “*Ca*. Parvarchaeum,” and 14 MAGs that form a new clade that is sister to Parvarchaeales, representing a new order according to the GTDB-based taxonomic assignments. We also reconstructed the metabolic potential and conducted in-depth comparative and evolutionary genomics analyses of these genomes, demonstrating their metabolic versatility and processes of adaptation to these extreme environments. With the reconstruction of several ancestral genomic features, we sought to examine the possible evolutionary trajectory regarding the adaptation of these lineages to different extreme environments.

## TAXONOMY

### Description of “*Candidatus* Jingweiarchaeum” gen. nov.

“*Candidatus* Jingweiarchaeum” (Jing.wei.ar.chae′um. Mandarin, *Jingwei*, a bird from ancient Chinese mythology who was originally the daughter of an ancient Chinese ruler, “Yan [Flame] Emperor,” and was drowned when playing alone on the seashore of the Eastern Sea; after her rebirth and becoming the “Jingwei” bird, albeit with her tiny figure, she became determined to fill up and defeat the ocean with wood and rocks piece by piece; N.L. neut. n. *archaeum*, archaeon, from Gr. adj. *archaios –ê –on*, ancient; N.L. neut. n. *Jingweiarchaeum*). The archaeon was named after Jingwei for her perseverance, which is similar to how DPANN archaea strive and survive in extreme environments despite their tiny cell sizes and limited metabolic capacities.

### Description of “*Candidatus* Jingweiarchaeum tengchongense” sp. nov.

“*Candidatus* Jingweiarchaeum tengchongense” (teng.chong.gen′se. N.L. neut. adj. *tengchongense*, referring to Tengchong county, Yunnan Province, China, where its first genome was reconstructed).

### Description of “*Candidatus* Jingweiarchaeaceae” fam. nov.

“*Candidatus* Jingweiarchaeaceae” (Jing.wei.ar.chae.a.ce′ae. N.L. neut. n. *Jingweiarchaeum*, a candidate genus; -*aceae*, ending to denote a family; N.L. fem. pl. n. *Jingweiarchaeaceae*, the *Jingweiarchaeum* candidate family).

### Description of “*Candidatus* Jingweiarchaeales” ord. nov.

“*Candidatus* Jingweiarchaeales” (Jing.wei.ar.chae.a′les. N.L. neut. n. *Jingweiarchaeum*, a candidate genus; -*ales*, ending to denote an order; N.L. fem. pl. n. *Jingweiarchaeales*, the *Jingweiarchaeum* candidate order).

### Description of “*Candidatus* Haiyanarchaeum” gen. nov.

“*Candidatus* Haiyanarchaeum” (Hai.yan.ar.chae′um. Mandarin, “*Haiyan*,” the husband of Jingwei who was said to be moved by her perseverance to fight with nature and joined the arduous journey to defeat the ocean. N.L. neut. n. *archaeum*, archaeon, from Gr. adj. *archaios –ê –on*, ancient; N.L. neut. n. *Haiyanarchaeum*).

### Description of “*Candidatus* Haiyanarchaeum thermophilum” sp. nov.

“*Candidatus* Haiyanarchaeum thermophilum” (ther.mo′phi.lum. Gr. fem. n. *therme*, heat; Gr. masc. adj. *philos*, loving; N.L. neut. adj. *thermophilum*, heat-loving; Haiyanarchaeum was found in sampling sites of ultrahigh temperatures, and potentially, this genus has characteristics of having high estimated optimal growth temperatures [OGTs]).

### Description of “*Candidatus* Haiyanarchaeaceae” fam. nov.

“*Candidatus* Haiyanarchaeaceae” (Hai.yan.ar.chae.a.ce′ae. N.L. neut. n. *Haiyanarchaeum*, a candidate genus; -*aceae*, ending to denote a family; N.L. fem. pl. n. *Haiyanarchaeaceae*, the *Haiyanarchaeum* candidate family).

### Description of “*Candidatus* Rehaiarchaeum” gen. nov.

“*Candidatus* Rehaiarchaeum” (Re.hai.ar.chae′um. N.L. neut. n. *archaeum*, archaeon; N.L. neut. n. *Rehaiarchaeum*, an archaeon from Rehai National Park, Tengchong, Yunnan Province, China).

### Description of “*Candidatus* Rehaiarchaeum fermentans” sp. nov.

“*Candidatus* Rehaiarchaeum fermentans” (fer.men′tans. L. part. adj. *fermentans*, fermenting; the novel species relies on fermentation for energy production, which is different from other lineages of Parvarchaeum).

### Description of “*Candidatus* Parvarchaeum tengchongense” sp. nov.

“*Candidatus* Parvarchaeum tengchongense” (teng.chong.en′se. N.L. neut. adj. *tengchongense*, referring to Tengchong County; the genome was recovered from Tengchong County, Yunnan Province, China).

All of the proposed names were registered in SeqCode (33).

## RESULTS AND DISCUSSION

### Quality and taxonomy hierarchy of the newly discovered genomes.

A total of 28 metagenome-assembled genomes (MAGs) were reconstructed from 28 metagenomes of geothermal spring sediment samples collected from January 2017 to August 2020 in Tengchong, Yunnan, China (see Tables S1 and S2 in the supplemental material). Three approaches were applied to evaluate the genome quality: (i) the frequency of occurrence of genes among 48 single-copy genes (SCGs) (see Table S3 for the counts of each SCG) (6, 17), (ii) genome quality assessed using the latest published CheckM2 package (34), and (iii) genome completeness and contamination estimated using CheckM, with the exclusion of markers that were universally lacking in the corresponding genus (Table S4) (31). All MAGs had low contamination (<5%). Twenty MAGs recovered in this study were of high quality (completeness of ≥90%, with the presence of both 16S and 23S rRNAs and >18 tRNAs), supported by at least one approach. The remaining MAGs were of medium quality, with a genome completeness of >50%. The results of taxonomy using GTDB-tk (35) demonstrated that 14 genomes were affiliated with the previously undescribed archaeal order “DTBS01” (named “*Candidatus* Jingweiarchaeales” here). Among them, seven MAGs were classified into a potential new genus, while the other seven MAGs were assigned to the undescribed genus “DTBS01,” with the proposed names “*Candidatus* Jingweiarchaeum” and “*Candidatus* Haiyanarchaeum” (see Taxonomy, above, and Table S1 in the supplemental material).

10.1128/msystems.01252-22.3 TABLE S1 General genome statistics, genome taxonomy, and sampling/predicted physiological characteristics of each organism represented by the associated genome. Download Table S1, XLSX file, 0.04 MB. Copyright © 2023 Rao et al. 2023 Rao et al. https://creativecommons.org/licenses/by/4.0/ This content is distributed under the terms of the Creative Commons Attribution 4.0 International license.

10.1128/msystems.01252-22.4 TABLE S2 Geochemical properties of sampling sites for all metagenomes. Download Table S2, XLSX file, 0.02 MB. Copyright © 2023 Rao et al. 2023 Rao et al. https://creativecommons.org/licenses/by/4.0/ This content is distributed under the terms of the Creative Commons Attribution 4.0 International license.

10.1128/msystems.01252-22.5 TABLE S3 Occurrences of 48 DPANN-specific single-copy genes (SCGs) present in 60 Parvarchaeales-related genomes. Download Table S3, XLSX file, 0.02 MB. Copyright © 2023 Rao et al. 2023 Rao et al. https://creativecommons.org/licenses/by/4.0/ This content is distributed under the terms of the Creative Commons Attribution 4.0 International license.

10.1128/msystems.01252-22.6 TABLE S4 Occurrences of 149 CheckM1 archaeal markers in 60 Parvarchaeales-related genomes. Download Table S4, XLSX file, 0.04 MB. Copyright © 2023 Rao et al. 2023 Rao et al. https://creativecommons.org/licenses/by/4.0/ This content is distributed under the terms of the Creative Commons Attribution 4.0 International license.

### Phylogenetic placement.

**FIG 1:**
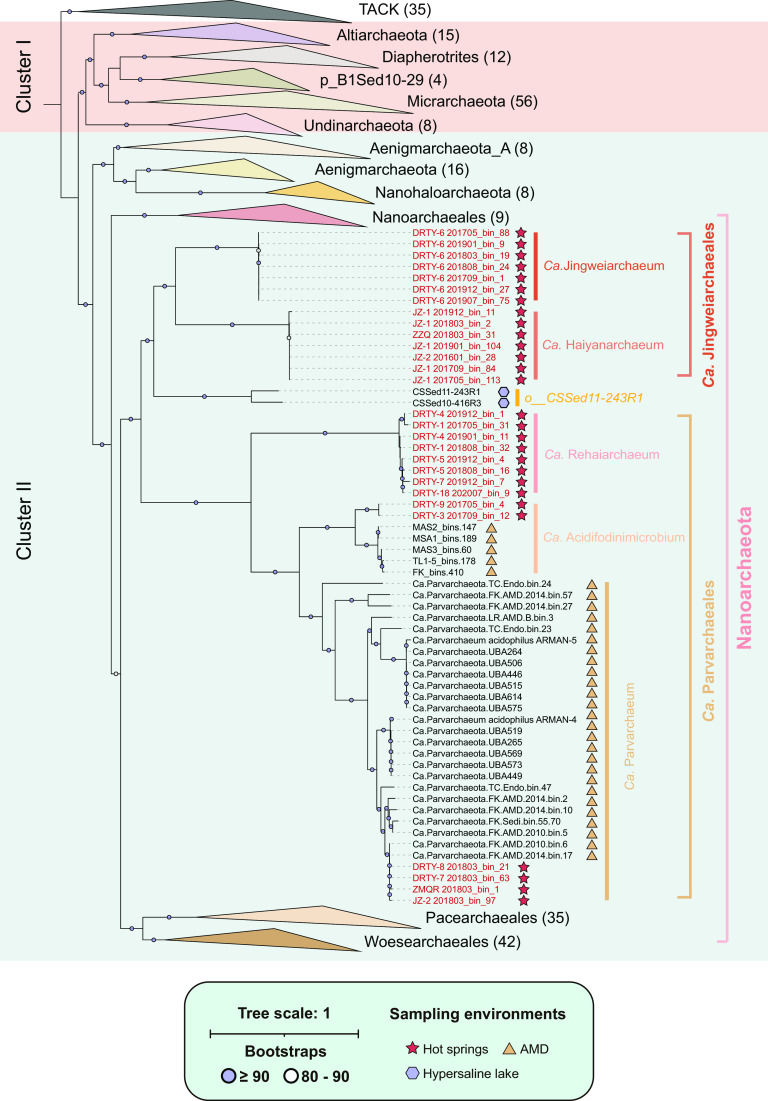
Phylogenetic placement of the newly discovered MAGs. The maximum likelihood tree was inferred from 53 concatenated archaeal proteins (32) (updated in GTDB r207 [https://gtdb.ecogenomic.org/stats/r207]) and generated via IQ-TREE v1.6.11 (93) with 1,000 ultrafast bootstrap iterations. Genomes recovered in this study are labeled in red on the tips of the phylogenetic tree. Sampling environments of the associated genomes are shown with red stars for hot springs, purple hexagons for hypersaline lakes, and yellow triangles for acid mine drainage (AMD). For bootstraps in each ancestral node, values of ≥90 are shown as purple solid circles, and values of between 80 and 90 are shown as hollow circles.

Consistent with results from a previous study (31), phylogenetic inference based on the concatenation of 53 conserved archaeal marker genes demonstrated that the DPANN superphylum was grouped into two clusters, DPANN cluster I and DPANN cluster II, with high bootstrap confidence values (≥90%) (Fig. 1; see also Table S5 for counts of all 53 markers in each genome). During phylogenetic construction, “*Candidatus* Huberarchaeota” genomes were excluded due to the possible long-branching attraction effect. When all MAGs from Parvarchaeales were considered, the phylogeny placed Huberarchaeota as the sister lineage of *Nanoarchaeota* (see Fig. S1a at https://doi.org/10.6084/m9.figshare.22126736.v3). We further tested if this was possibly due to the uneven selection of representative genomes of these lineages (36). However, when these closely related MAGs were dereplicated using dRep (37) with a 99% average nucleotide identity (ANI) as the cutoff, the phylogenetic position of *Nanoarchaeota* remained unstable after selection using dRep (see Fig. S1b at https://doi.org/10.6084/m9.figshare.22126736.v3). Specifically, Huberarchaeota branched within the “*Nanoarchaeales*” with low bootstrap confidence at their parent node. We further sought to examine if this was caused by the potential long-branch attraction (LBA) effect of the Huberarchaeota genomes since they presented a deep-rooted long branch in previous studies (31, 38). After the exclusion of Huberarchaeota genomes, the topology structure became stable, and each node within *Nanoarchaeota* is well supported, with a bootstrap confidence value of ≥80 (Fig. 1). Two reference genomes, which were previously labeled Parvarchaeota in NCBI Taxonomy and order CSSed11-243R1 in GTDB, were shown to form a sister lineage with Jingweiarchaeales. Within the phylum *Nanoarchaeota*, *Nanoarchaeales* represent the deepest-rooted lineage, while Jingweiarchaeales, CSSed11-243R1, and Parvarchaeales together form a sister lineage with *Woesearchaeales* and *Pacearchaeales*.

10.1128/msystems.01252-22.7 TABLE S5 Occurrences of 53 GTDB archaeal markers in all genomes selected for phylogenetic analyses. Download Table S5, XLSX file, 0.07 MB. Copyright © 2023 Rao et al. 2023 Rao et al. https://creativecommons.org/licenses/by/4.0/ This content is distributed under the terms of the Creative Commons Attribution 4.0 International license.

### Environmental distribution.

We found that both Jingweiarchaeales and Parvarchaeales present low relative abundances in the associated sampling environments (<0.5% for Jingweiarchaeales and <4% for Parvarchaeales) (see Fig. S2 at https://doi.org/10.6084/m9.figshare.22126736.v3). A previous pioneering study revealed the limited distribution of Parvarchaeales, which were detected only in acidic environments such as AMD and a Tengchong acidic endolithic community (9). This led to the question of whether the Parvarchaeales are an acidophilic lineage that specifically inhabits acidic environments. In this study, we found that most of the available Parvarchaeum genomes were retrieved from extremely acidic environments with pH <3 and thus might represent an acidophilic archaeal lineage (Table S1; see also Fig. S2 at https://doi.org/10.6084/m9.figshare.22126736.v3) (39). This inference was confirmed by the observation for our samples that Parvarchaeales often appeared at higher relative abundances (>1%) when the pH was <4 and increased over time in site DRTY-6 as the pH decreased (see Fig. S2 at https://doi.org/10.6084/m9.figshare.22126736.v3).

### Metabolic potentials.

**FIG 2:**
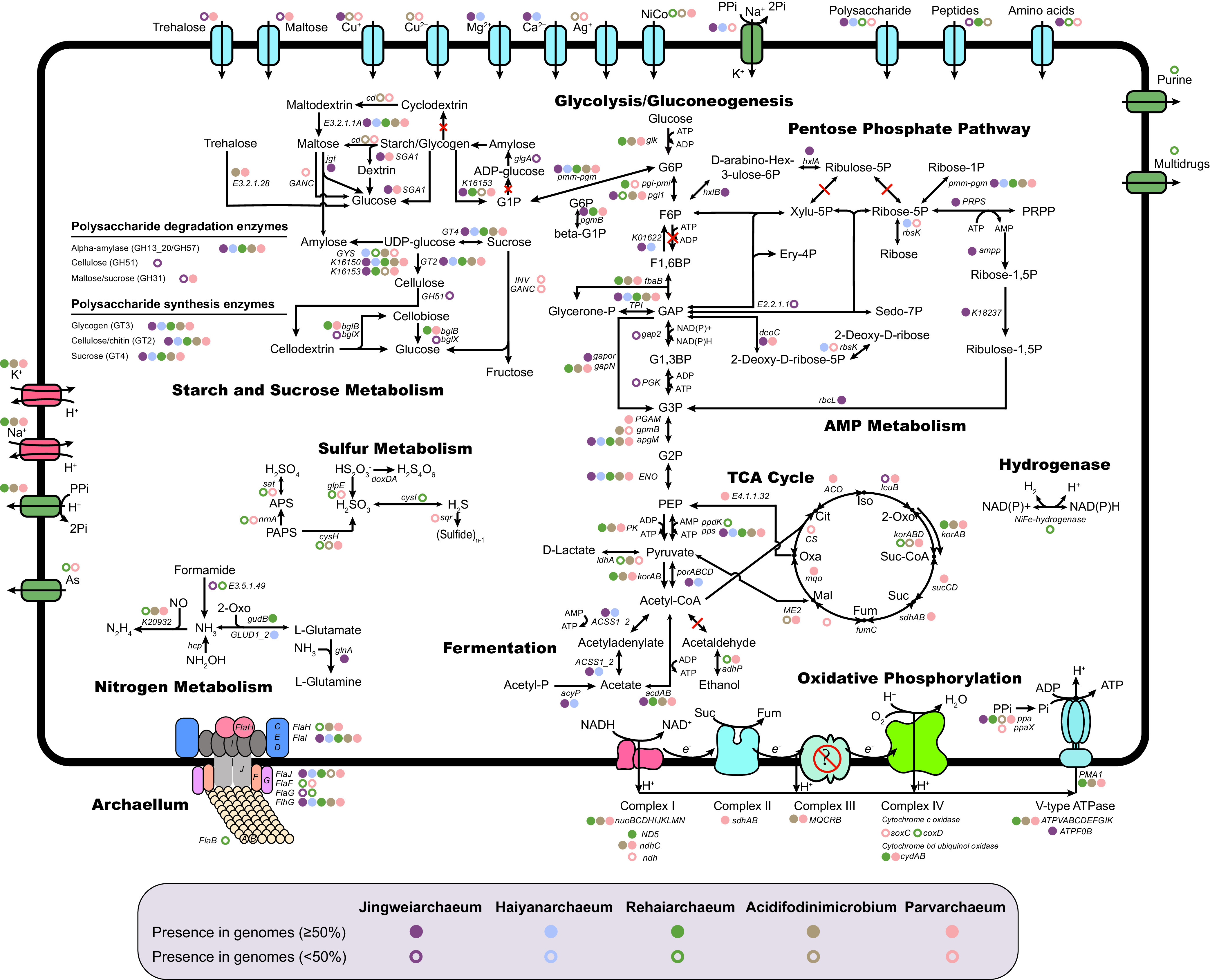
Metabolic pathways in Jingweiarchaeum, Haiyanarchaeum, Rehaiarchaeum, Acidifodinimicrobium, and Parvarchaeum that were recovered from 60 MAGs. The presence of genes in Jingweiarchaeum, Haiyanarchaeum, Rehaiarchaeum, Acidifodinimicrobium, and Parvarchaeum are shown with dark-red, blue, green, yellow, and pink symbols, respectively. Genes that are present in >50% of the genomes are illustrated with solid circles, while genes that are present in <50% of the genomes but in at least one MAG are shown with hollow circles. The names of genes are labeled next to the circles representing the presence of genes. G6P, glucose-6-phosphate; F6P, fructose-6-phosphate; F1,6BP, fructose-1,6-bisphosphate; GAP, glyceraldehyde-3-phosphate; Glycerone-P, glycerone-phosphate; G1,3BP, glycerate-1,3-bisphosphate; G3P, glycerate-3-phosphate; G2P, glycerate-2-phosphate; PEP, phosphoenolpyruvate; Xylu-5P, xylulose-5-phosphate; Ribulose-5P, ribulose-5-phosphate; Ribose-5P, ribose-5-phosphate; Sedo-7P, sedoheptulose-7-phosphate; Ery-4P, erythrose-4-phosphate; Ribose-1P, ribose-1-phosphate; PRPP, phosphoribosyl diphosphate; Ribose-1,5P, ribose-1,5-bisphosphate; Ribulose-1,5P, ribulose-1,5-bisphosphate; 2-Deoxy-d-ribose-5P, 2-deoxy-d-ribose 5-phosphate; 2-Deoxy-d-ribose, 2-deoxy-d-erythro-pentose; d-arabino-Hex-3-ulose-6P, d-arabino-3-hexulose 6-phosphate; G1P, glucose-1-phosphatase; Oxa, oxaloacetate; Cit, citrate; Iso, isocitrate; 2-Oxo, 2-oxoglutarate; Suc-CoA, succinyl-CoA; Suc, succinate; Fum, fumarate; Mal, malate; APS, adenosine 5′-phosphosulfate; PAPS, 3′-phosphoadenosine 5′-phosphosulfate; TCA cycle, tricarboxylic acid cycle.

By reconstructing the metabolic pathways of Jingweiarchaeales and Parvarchaeales (Fig. 2 and Table S6), Jingweiarchaeum is the only genus with the detection of a complete AMP that was commonly observed in DPANN lineages. Phylogenetic inference identified a type III *rbcL* gene encoding the large subunit of ribulose 1,5-bisphosphate carboxylase/oxygenase (RuBisCO) in Jingweiarchaeum, consolidating the potential function involved in the nucleotide salvage pathway (see Fig. S3 at https://doi.org/10.6084/m9.figshare.22126736.v3) (40–42). The detection of a partial pentose phosphate pathway (PPP) in Jingweiarchaeum microbes suggested that they could convert fructose-6-phosphate (F6P) and glyceraldehyde-3-phosphate (GAP) into phosphoribosyl diphosphate (PRPP). Given the absence of glucokinase and fructose-1,6-biphosphate kinase (F1,6BP) in glycolysis, the partial PPP coupling with AMP enables the conversion of glycerate-3-phosphate (G3P), which further leads to the generation of pyruvate and ATP by linking to glycolysis. The TCA cycle and the electron transfer chain (ETC), such as the V-type ATPase complex, are beyond detection, suggesting that Jingweiarchaeum may rely heavily on fermentation for energy production. As expected, Jingweiarchaeum might be able to ferment acetate by harboring acetyl-CoA synthetase (ADP forming) and acetyl-CoA synthetase encoded by *acdAB* and ACSS1_2 (43). In contrast, Haiyanarchaeum microbes possess an even more reduced glycolytic pathway and lack PPP and AMP. In addition, genes associated with acetate fermentation are also missing, suggesting a minimal metabolic capacity that is similar to that of “*Candidatus* Woesearchaeota” archaeon AR20, suggesting that Haiyanarchaeum microbes may be obligate symbionts and rely heavily on their hosts for resources (6, 13).

10.1128/msystems.01252-22.8 TABLE S6 Summary of the metabolic potentials of 58 Jingweiarchaeales and Parvarchaeales genomes. Download Table S6, XLSX file, 0.08 MB. Copyright © 2023 Rao et al. 2023 Rao et al. https://creativecommons.org/licenses/by/4.0/ This content is distributed under the terms of the Creative Commons Attribution 4.0 International license.

Consistent with the results of a previous study, Parvarchaeum (formerly “*Ca.* Parvarchaeota”) MAGs encode nearly complete pathways for glycolysis, the TCA cycle, and the ETC (9). However, we were unable to detect phosphofructokinase in all Parvarchaeum MAGs, including genomes described in previous research (9). It is unclear whether this is due to annotations with different databases or changes in the associated databases that have altered the previous annotation results. On the contrary, most genes associated with the TCA cycle and the ETC were missing in Rehaiarchaeum and “*Ca*. Acidifodinimicrobium,” indicating that they might rely on acetate fermentation for energy production with the presence of *acdAB* genes (44). Besides, Rehaiarchaeum and Parvarchaeum possess genes encoding alcohol dehydrogenases (*adhP*), enabling them to ferment ethanol. However, none of these MAGs have aldehyde dehydrogenase for the conversion of acetyl-CoA into aldehyde, indicating that they might rely on their hosts to promote the fermentation process.

The presence of only one menaquinol-cytochrome *c* reductase cytochrome *b* subunit (MQCRB) in Acidifodinimicrobium and Parvarchaeum genomes raises the question of whether these genomes indeed encode fully functional complex III of the ETC. Similarly, only two subunits, *coxD* and *soxC*, involved in complex IV were detected in a few Parvarchaeum MAGs. However, both Rehaiarchaeum and Parvarchaeum have *cydAB* genes encoding cytochrome *bd* ubiquinol oxidase, which were reported to have high oxygen affinity and were expressed under oxygen-limited conditions (45). The cytochrome *bd* ubiquinol oxidase could use ubiquinol as an electron carrier coupled with the reduction of oxygen and could generate proton motive force (PMF), possibly in place of the function of complexes III and IV and consuming extra oxygen to maintain low-oxygen intracellular environments (46, 47). All of these results suggest that although having a complete TCA cycle and a potentially complete ETC, Parvarchaeum may be oxygen tolerant and may inhabit environments with lower levels of oxygen rather than favoring aerobic environments. However, we could not rule out the possibility that some DPANN archaea may acquire the ability to respire oxygen using the ETC coupled with the TCA cycle, or at least they may use the PMF of their potential hosts when in proximity to complex V (13).

**(i) Gluconeogenesis, polysaccharide degradation, and biosynthesis.** Although Parvarchaeales lack a complete glycolysis pathway, Jingweiarchaeales MAGs appear to carry genes for the gluconeogenesis pathway with G6P as the end product, including *porABCD*, *pps*, and K01622. The ancestral gluconeogenic enzyme fructose 1,6-bisphosphate (FBP) aldolase/phosphatase, exhibiting both FBP aldolase and FBP phosphatase activities, was detected. It has been reported that the FBP phosphatase is irreversible, which renders gluconeogenesis unidirectional (48). The production of G6P via gluconeogenesis might further be connected to the synthesis of polysaccharides by the further conversion of G6P to glucose-1-phosphatase (G1P) by *pmm-pgm* genes encoding phosphomannomutase/phosphoglucomutase. Functional annotation results based on comparison to the Carbohydrate-Active Enzymes (CAZy) database (49) revealed that both Jingweiarchaeales and Parvarchaeales are capable of synthesizing glycogen (β-glucosidase [GT3]), cellulose (β-glucosidase [GT2]), and sucrose (sucrose synthase [GT4]). Besides, all genera contain genes encoding glycogen degradation, including GH13_20 and/or GH57. The detection of SGA1 and *jgt* genes, encoding glucoamylase and 4-α-glucanotransferase, in Jingweiarchaeum suggests that this genus is capable of degrading maltose with the release of glucose. It seems plausible that maltose can serve as an intermediate of starch/glycogen degradation, which leads to the final production of monosaccharides. This could be further exemplified by the observation of GH31 in Jingweiarchaeum MAGs. In addition, Jingweiarchaeum can also degrade cellulose with GH51, demonstrating its versatility in polysaccharide utilization. The presence of trehalose and maltose transport systems, as well as genes associated with their degradation via α,α-trehalase (EC 3.2.1.28), in Parvarchaeum suggests that external import may serve as an alternative source of sugars to sustain the glycolysis pathway in this genus. Taken together, we conjectured that the detected gluconeogenesis and polysaccharide biosynthesis pathways in Jingweiarchaeales, especially Jingweiarchaeum, might contribute to the storage of polysaccharides under nutrient-rich conditions. Both of these DPANN archaea and putative hosts may gain ATP from these monosaccharides to sustain their growth through the glycolytic pathway or the above-mentioned coupled pathways of partial glycolysis, AMP, and PPP.

**(ii) Cell membrane biosynthesis.** All three genera in Parvarchaeales lack genes to biosynthesize isoprenoid precursor isopentenyl pyrophosphate (IPP) and the phospholipid backbone, the main constituents of the cell membrane. Similarly, the related genes also failed to be detected in most Jingweiarchaeales MAGs, except for the *araM* gene, which encodes glycerol-1-phosphate dehydrogenase [NAD(P)^+^] (G1PDH). To our surprise, each Jingweiarchaeum MAG possesses an *mvk* gene that encodes mevalonate kinase, which is vital for the biosynthesis of the archaeal membrane precursor IPP. However, the biosynthetic pathway of IPP and further to archaeol were incomplete in Jingweiarchaeum due to the lack of genes encoding phosphomevalonate kinase (EC 2.7.4.2), diphosphomevalonate decarboxylase (*mvd*), and archaetidylserine synthase (*pssA*). Haiyanarchaeum genomes, on the other hand, have even more genes missing in the associated pathways. Consequently, they must rely on their hosts to provide key intermediates to assist in the biosynthesis of archaeal cell membranes. Although the *glpA* gene encoding glycerol-3-phosphate dehydrogenase (G3PDH) was surprisingly found in all genera of Parvarchaeales, they are unable to synthesize a bacterial cell membrane since most genes associated with the methylerythritol phosphate (MEP) pathway and phospholipid biosynthesis were absent. All of these results indicated the host dependence of Jingweiarchaeales and Parvarchaeales.

The archaellum and pilus are considered to be associated with the motility, attachment, and biofilm formation of archaea (50). Several previous studies have demonstrated that DPANN archaea can probably penetrate their host cells via pili or pilus-like structures (17, 51). We found that besides the *flaHIJ* genes encoding the archaellum base structure, the *flaB* gene encoding the major archaellin and other genes were found in one Rehaiarchaeum MAG, DRTY-4_201901_bins_11. Besides, genes encoding pilin (*pilA* subfamily; arCOG02420 for Jingweiarchaeum and arCOG03871 for Haiyanarchaeum) were detected in all genomes, as were genes encoding other structural proteins in some of the MAGs within Jingweiarchaeales and Parvarchaeales (52).

**(iii) Hydrogen, sulfur, and nitrogen metabolism.** One Rehaiarchaeum MAG, DRTY-1_201705_bins_31, possesses NiFe-hydrogenase encoded by *hyaAB* as well as several hydrogenase maturation and formation proteins. The absence of hydrogenase indicates that most members of the Jingweiarchaeales and Parvarchaeales rely on alternative strategies for NAD(P)H and NAD(P)^+^ interconversion. Unlike most DPANN archaea, Parvarchaeales genomes harbor several genes related to sulfur and nitrogen metabolism. Specifically, Rehaiarchaeum genomes harbor the *cysI* gene encoding sulfite reductase (NADPH) (53). The reversible reaction may result in the interconversion of NAD(P)^+^ and NAD(P)H in place of the limited hydrogenases, which may be coupled with their organic carbon oxidation in both partial glycolysis and acetate/ethanol fermentation. Strikingly, genes encoding hydrazine synthase (K20932; EC 1.7.2.7) were found in all three genera within Parvarchaeales, which have never been found in any DPANN archaea. As reported previously, this enzyme participates in anaerobic ammonium oxidation by the production of highly reactive hydrazine from ammonia and nitric oxide, which could be further reduced to N_2_ (54, 55). However, the exact role of this gene in Parvarchaeales still needs to be experimentally verified.

### Comparative genomics analysis of adaptabilities to various habitats.

To ensure the accuracy of genome statistics and comparative genomics, only genomes in this study and reference genomes with ≥80% completeness determined by both 48 SCGs and CheckM2 were kept for further analyses. However, with the assessment of genome quality by CheckM2 and the frequency of occurrence of markers among 48 SCGs, we observed that the Rehaiarchaeum and Acidifodinimicrobium MAGs collectively have low genome qualities. Thus, we further examined if there were other markers universally missing in these lineages. Apart from PF01849.13, PF01912.13, PF01922.12, PF04127.10, PF05221.12, PF06026.9, TIGR00336, TIGR00670, and TIGR01213, which were consistently absent in Jingweiarchaeales and Parvarchaeales, several other markers were also missing within each specific genus. For example, the three genera in Parvarchaeales lack PF13685.1, TIGR03677, and TIGR00432, while they all can be detected in Jingweiarchaeales. We further modified the 149 archaeal markers used by CheckM1 by excluding markers that were universally missing in each genus, which included 11 markers missing in Jingweiarchaeum, 21 markers missing in Haiyanarchaeum, 29 markers missing in Rehaiarchaeum, 21 markers missing in Acidifodinimicrobium, and 17 markers missing in Parvarchaeum, to assess the genome quality of each MAG (Table S5). By applying this approach, 48 MAGs with a completeness of ≥80% were kept for further comparative genomics analysis (Table S1). Due to the lack of complete genomes, however, we still cannot rule out the possibility that technical biases during sequence assembly and genome binning may have led to missing markers.

**FIG 3:**
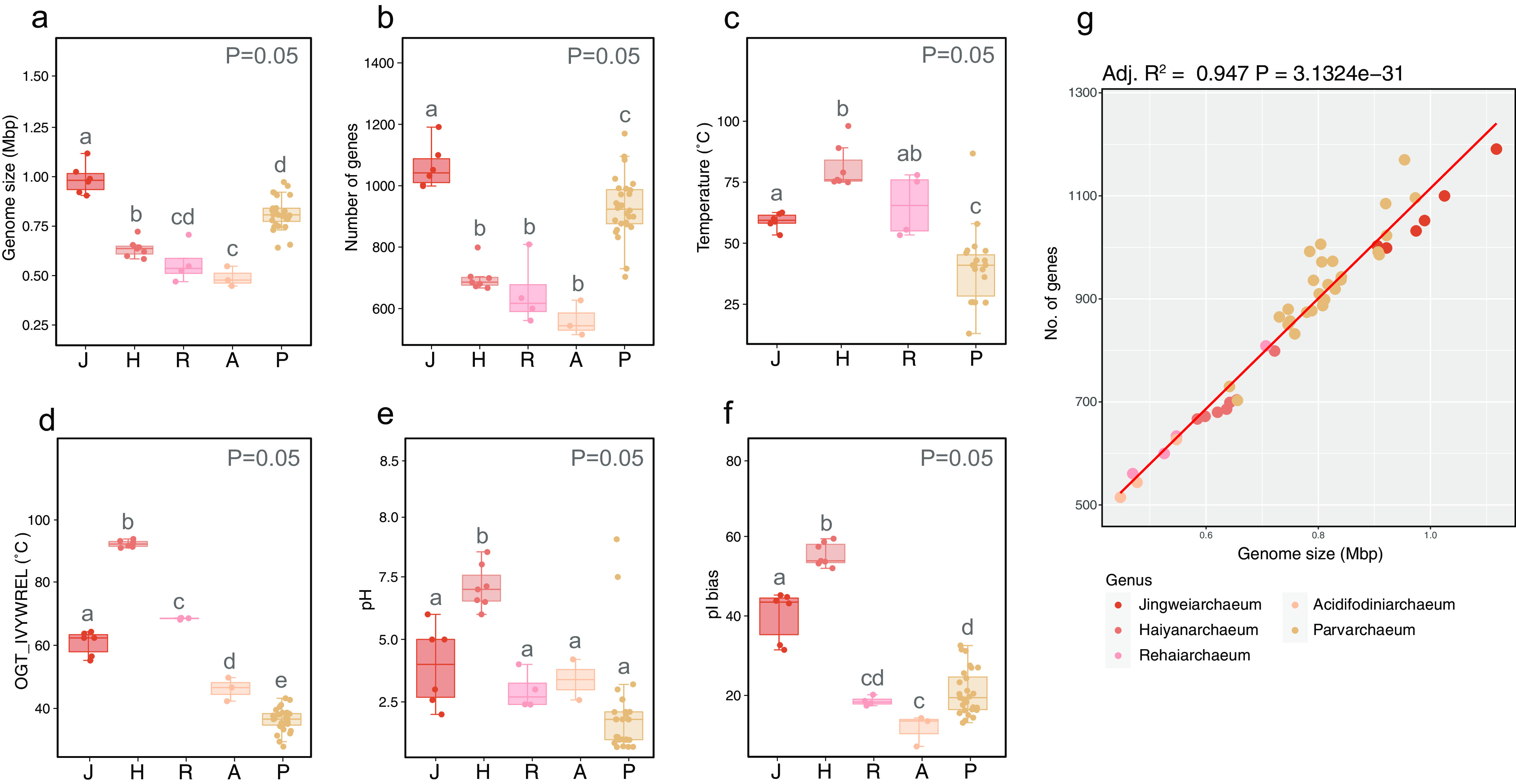
Genomic comparisons of Jingweiarchaeum (J), Haiyanarchaeum (H), Rehaiarchaeum (R), Acidifodinimicrobium (A), and Parvarchaeum (P). Differences in genome sizes (a), numbers of genes (b), temperatures (c), OGTs predicted by the overrepresentation of 7 amino acids (IVYWREL) (d), pHs (e), average isoelectric points (pIs) (f), and the corelation of number of protein-coding genes and genome sizes of the five genera (g) were visualized using the ggplot2 package (v3.3.6) (100). Genomic feature differences were tested by the Wilcoxon signed-rank test using the wilcox.test() function in the stat package (v4.1.1) (99) in R. Different letters show significant differences between groups with a *P* value of <0.005.

To avoid the bias of genomic feature statistics caused by incomplete genome assemblies, we examined the effect of genomic completeness on the relative genome size differences in all genera in this study, which showed stability over different approaches (Fig. 3a; see also Fig. S4a to c at https://doi.org/10.6084/m9.figshare.22126736.v3). Overall, Jingweiarchaeum MAGs have the largest genomes among all five genera, followed by Parvarchaeum, while Rehaiarchaeum MAGs have the smallest genomes. This is significantly better explained by the number of protein-coding sequences (CDSs), where a significant positive relationship was observed between the genome sizes and the numbers of CDSs (adjusted *R*^2^ = 0.94; *P* = 3.1324e−31) (Fig. 3b and g). The genomes of Haiyanarchaeum appear to be the most compact, with small genomes, high coding densities, and the highest ratios of overlapping genes (see Fig. S4e and f at https://doi.org/10.6084/m9.figshare.22126736.v3). The high compactness of these genomes might be a result of adaptation to environments with high temperatures (Fig. 3c) (56). Regarding the GC contents of the genomes of these genera, they vary across lineages (see Fig. S4g at https://doi.org/10.6084/m9.figshare.22126736.v3).

Principal-coordinate analyses (PCoAs) based on the metabolic profiles of KEGG and arCOG annotation results demonstrated that the genomes were well clustered based on their phylogenetic positions rather than habitats (see Fig. S5 at https://doi.org/10.6084/m9.figshare.22126736.v3). The short distance between Acidifodinimicrobium and Parvarchaeum suggests that a recent geographical dispersion possibly occurred. This is also supported by the average amino acid identities (AAIs) between these lineages (see Fig. S6 at https://doi.org/10.6084/m9.figshare.22126736.v3). The larger sizes of Parvarchaeum genomes within the PCoA plots exhibit higher genomic plasticity among these genera, which may give them the ability to better adapt to different habitats. However, the clustering of Parvarchaeum genomes from both high- and low-temperature environments suggests that the adaptation to different environments may not yet have induced notable genomic shifts in time. This inference can be further corroborated by the observation of wider ranges of GC contents, isoelectric points (pIs), and estimated optimal growth temperatures (OGTs) in this genus, which demonstrates a more diverse nucleotide/amino acid composition (Fig. 3d and f; see also Fig. S4g to j at https://doi.org/10.6084/m9.figshare.22126736.v3). In contrast, Jingweiarchaeum, Haiyanarchaeum, and Rehaiarchaeum inhabit primarily thermal habitats, whereas Acidifodinimicrobium and Parvarchaeum favor environments with lower temperatures.

**(i) Adaptation to high-temperature environments.** The investigation of sampling sites suggested that Haiyanarchaeum genomes were derived mostly from samples from environments with higher temperatures, while Parvarchaeum genomes were from samples from environments with lower temperatures (Fig. 3c). Overall, Haiyanarchaeum microbes appear to be hyperthermophilic, with the OGTs of >80°C predicted by IVYWREL (Fig. 3d). Expectedly, *rgy* genes, encoding reverse gyrase that was ubiquitously present in hyperthermophiles, were detected in the genomes of Haiyanarchaeum, further suggesting that they might indeed represent a hyperthermophilic lineage (Table S6) (57–59). Given the exclusive detection of currently available Jingweiarchaeum MAGs, we infer that Jingweiarchaeum microbes might also be thermophiles. Most MAGs within Parvarchaeum and Acidifodinimicrobium are mesophiles, for which growth temperatures of <50°C are preferable.

However, when looking into other genes previously considered vital for thermal adaptation (60), they did not show specificity to Jingweiarchaeales, which had higher OGTs and were sampled from sites with higher temperatures (Table S6). These genes encoding heat shock proteins (HSPs), chaperones, and prefoldins, which can protect proteins from denaturation, were widely detected among all MAGs (61). Besides, the possession of the DNA repair protein RadA that mediates the recombination process (62), topoisomerases that maintain DNA structure, and polyamines such as spermidines (*speE*) that stabilize the cytoplasm also confer the ability to resist high-temperature stress (61). Besides thermal adaptation, these genes can also allow survival under other stresses such as acid stress. The equivalent distributions of these genes among Jingweiarchaeales and Parvarchaeales MAGs strengthen this inference. It is noteworthy that Jingweiarchaeales genomes uniquely harbor DNA repair systems encoded by ERCC4, *ykoV*, and *splB*. However, we could not confirm whether they were exploited to cope with high-temperature stress.

The predicted OGTs in this study largely reflect the preference of amino acid usage for each genome since all methods consider only or partly the amino acid compositions of proteomes for prediction. As expected, the accumulation of glutamate (Glu) (E) was observed in the genomes of Jingweiarchaeum, Haiyanarchaeum, and Rehaiarchaeum (see Fig. S7 at https://doi.org/10.6084/m9.figshare.22126736.v3), in particular Haiyanarchaeum, also with more representation of arginine (Arg) (R). Previous studies have shown that a higher fraction of Arg residues could enhance thermostability with more stable salt bridges (63), among which the combination of arginine and glutamate (R+E) was shown to be the strongest (64). The accumulation of proline could also potentially decrease the flexibility of the protein secondary structure as well as the efficiency of proline isomerization, which may also increase the thermostability of proteins (65–67). As a result, the higher fractions of R+E and P within Haiyanarchaeum proteomes may be vital to their adaptation to higher-temperature environments. Collectively, the adaptation strategies of Jingweiarchaeales, especially hyperthermophilic Haiyanarchaeum, may include genome streamlining with small genomes, high coding densities, high ratios of overlapping genes, and biases of amino acid usage that favor high-temperature environments.

**(ii) Adaptation to acidic environments.** Many MAGs from Parvarchaeales in this study were recovered from acidic environments such as AMD and acid hot springs. Given this, we further examined the pI of each genome to reveal the intracellular pH (68, 69). Interestingly, all genomes, regardless of the pH of their samples, exhibit bimodal distributions, with a relatively low fraction of proteins with a pI close to neutral pH (see Fig. S8 to S13 at https://doi.org/10.6084/m9.figshare.22126736.v3). This is expected to be a result of the instability and insolubility of proteins at a pH close to the pI (70). Furthermore, as shown by the pI bias of these genomes, none of these genomes were overrepresented by acidic proteins compared to basic proteins (pI bias of >0), demonstrating that the inner cell environments of these archaea were nearly neutral (Fig. 3f; see also Fig. S4h at https://doi.org/10.6084/m9.figshare.22126736.v3). Unlike most halophilic archaea, which sustain a low acidic intracellular pH to improve amino acid solubility (70), the maintenance of a nearly neutral intracellular pH (as shown by the nearly neutral pI) suggested that Parvarchaeales tend to be acid-tolerant microbes rather than acidophiles. This inference is further supported by the detection of a P-type H^+^-exporting transporter (PMA1) and an Na^+^/H^+^ antiporter (NhaP) in Parvarchaeales genomes, which could potentially export excess protons extracellularly to alleviate acid stress (Fig. 2 and Table S6) (47, 71). Interestingly, Jingweiarchaeum genomes appear to have different strategies to cope with acidic stress, including the decarboxylase of organic acids (*speA*, *mfnA*, and *pdaD*) and the formation of a proton-impermeable membrane by the synthesis of hopanoid (*sqhC*) to maintain intracellular pH homeostasis (39). The *trkA*-encoded Trk/Ktr system potassium uptake proteins were detected in both Jingweiarchaeum and all Parvarchaeales genera. This system was shown to facilitate the import of potassium ions and may inhibit proton influx by creating a chemiosmotic barrier (39, 72).

Oxidative stress appears to be another obstacle to survival in acidic environments (73). As mentioned above, most MAGs within the Parvarchaeales harbor the high-oxygen-affinity cytochrome oxidase *cydAB* genes, which might be associated with the removal of oxygen (45–47). Besides, other genes, including superoxide dismutase (*sodA*) and peroxiredoxin (*bcp* and *ahpC*) genes, were found solely in all genera of Parvarchaeales. These genes guarantee the physiological activity of these microbes when they are exposed to oxic environments.

### Origin and evolution of Jingweiarchaeales and Parvarchaeales lineages.

**FIG 4:**
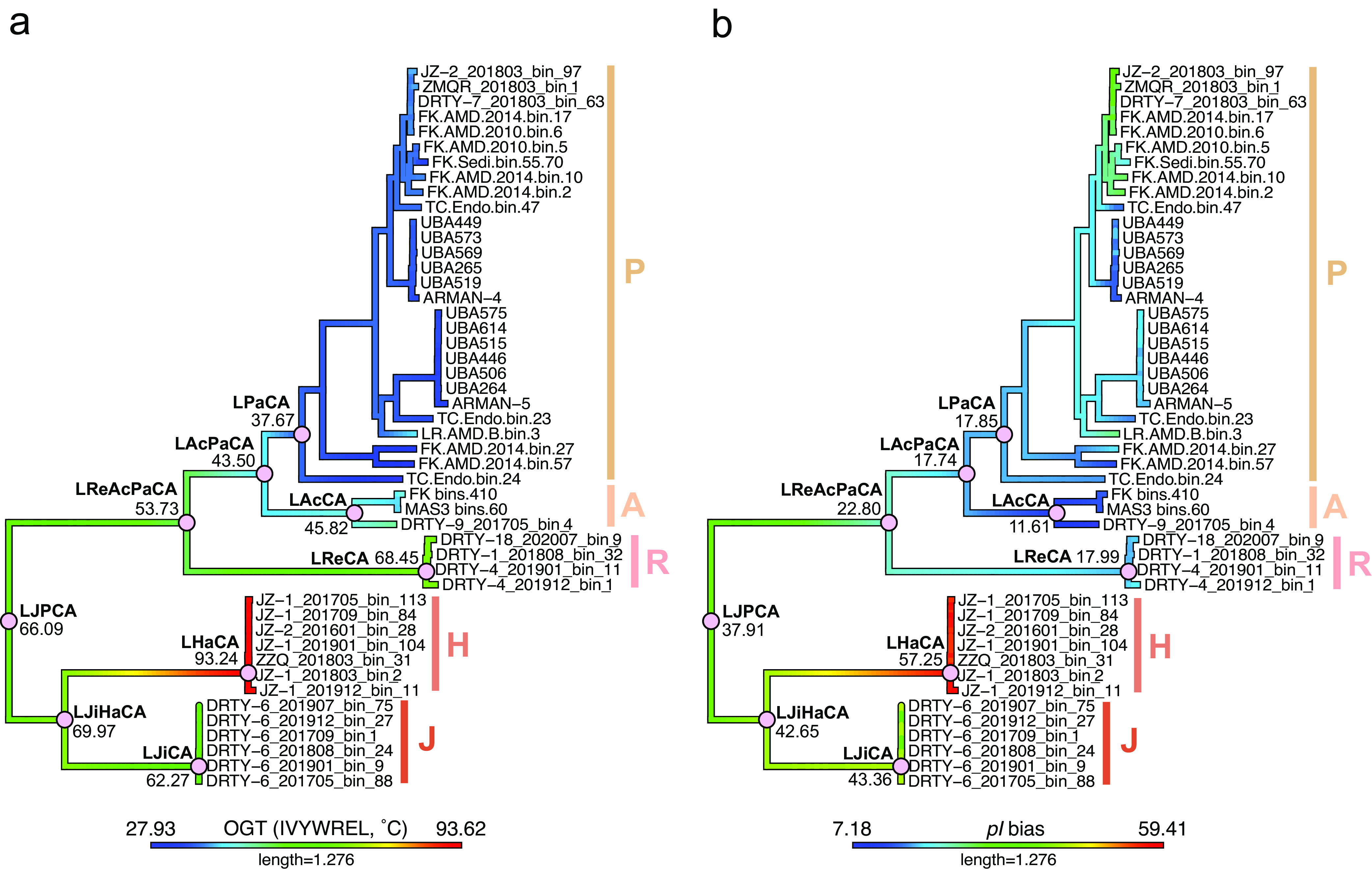
Inference of ancestral traits of Jingweiarchaeales and Parvarchaeales, including the predicted optimal growth temperature (OGT) and isoelectric point (pI). The evolutionary changes in OGTs predicted by IVYWREL (a) and pI bias (b) from the last common ancestor of Jingweiarchaeales and Parvarchaeales (LJPCA) are shown, along with the topology of the phylogeny placement in Fig. 1. The reconstruction of ancestral traits was conducted using the fastAnc() function in the phytools package (v1.2-0) in R (102). The nodes of the inferred last common ancestors are marked by pink solid circles, with the predicted genomic feature of the last common ancestor labeled below. Abbreviations: LJiHaCA, last common ancestor of Jingweiarchaeum and Haiyanarchaeum; LReAcPaCA, last common ancestor of Parvarchaeum, Acidifodinimicrobium, and Rehaiarchaeum; LJiCA, last common ancestor of Jingweiarchaeum; LHaCA, last common ancestor of Haiyanarchaeum; LAcPaCA, last common ancestor of Acidifodinimicrobium and Parvarchaeum; LPaCA, last common ancestor of Parvarchaeum; LAcCA, last common ancestor of Acidifodinimicrobium; LReCA, last common ancestor of Rehaiarchaeum. The color bar below each panel illustrates the color ranges corresponding to the values for each trait.

By applying three approaches to calculate the OGTs of all MAGs in this study, we observed that three genera, Jingweiarchaeum, Haiyanarchaeum, and Rehaiarchaeum, are more likely to inhabit thermal environments (Fig. 3c and 3d; see also Fig. S4i and j at https://doi.org/10.6084/m9.figshare.22126736.v3). With Jingweiarchaeales and the deep-rooted lineages of Parvarchaeales showing high OGTs, we hypothesized that these lineages possibly originated from high-temperature environments. This inference is well supported by the OGT predictions of ancestral nodes. OGTs predicted by IVYWREL revealed that the last common ancestor of Jingweiarchaeales and Parvarchaeales (LJPCA) had an OGT of 66.09°C (Fig. 4a). After the differentiation of the LJPCA, the OGTs tended to decrease toward the evolution of Parvarchaeum but with an increasing pattern in Jingweiarchaeum. The last common ancestors of Parvarchaeum (LPaCA) and Haiyanarchaeum (LHaCA) may have thrived at OGTs of 37.67°C and 93.24°C, respectively. In contrast, the OGTs of the last common ancestors of Jingweiarchaeum (LJiCA) and Rehaiarchaeum (LReCA) remained relatively unchanged compared to that of the LJPCA. Collectively, both phylogenetic placements and OGT predictions of ancestral nodes support the hot origins of the two orders. Further evolution resulted in the adaptation of Parvarchaeum to mesophilic environments and the emergence of hyperthermophilic Haiyanarchaeum.

To uncover the amino acid usage patterns across the two orders, the ancestral-state reconstruction of pI and pI bias was estimated (Fig. 4b; see also Fig. S4h at https://doi.org/10.6084/m9.figshare.22126736.v3). Generally, all leaves and ancestral nodes showed neither low average pI nor low pI bias values, suggesting that they sustain a circumneutral intracellular pH. Although all nodes span a narrow range of pIs, we still observe an increasing pattern from the LJPCA to the LJiCA but the opposite trend from the LJPCA to the LPaCA. The variation is more notable with regard to pI bias calculations. To cope with acid stress and manage a circumneutral intracellular pH, different strategies have been adopted. As mentioned above, Parvarchaeales tend to purge protons with proton efflux systems, while Jingweiarchaeales favor the utilization of buffer molecules to maintain intracellular pH homeostasis.

Analyses of the metabolic potentials of Jingweiarchaeales and Parvarchaeales have shown clear niche differentiation between them. Within the Jingweiarchaeales, most microbes are strict anaerobes with fermentation and substrate salvage capacities. Particularly, Jingweiarchaeum genomes harbor an incomplete “rerouted glycolysis” pathway that utilizes the PPP for intermediate conversion and a complete AMP pathway for energy generation. Besides, they are able to obtain energy through acetate fermentation. In contrast, the genomes of Haiyanarchaeum are more streamlined (Fig. 3a; see also Fig. S4e and f at https://doi.org/10.6084/m9.figshare.22126736.v3), with the absence of most genes for glycolysis, AMP, and the PPP, suggesting that they have to depend on hosts to provide the necessary substrates for energy generation. All members of Haiyanarchaeum represent hyperthermophiles due to the wide detection of *rgy* genes. Further investigation of these genes demonstrates that the hyperthermophilic features of Haiyanarchaeum were acquired from the *Crenarchaeota* via horizontal gene transfer (HGT) (see Fig. S14 at https://doi.org/10.6084/m9.figshare.22126736.v3). We reasoned that temperature may lead to the differentiation of these two genera and that higher temperatures may result in more streamlined genomes by the loss of more nonessential genes.

Parvarchaeales seem to inhabit habitats with relatively low temperatures, although some of the lineages may originate from thermal environments (i.e., Rehaiarchaeum) (Fig. 4a). Unlike Jingweiarchaeales, which rely on fermentation to gain energy, all genera of Parvarchaeales harbor a complete or nearly complete glycolysis pathway. Within each order, it seems that the higher the temperature of the environment that the microbes inhabit, the smaller their genomes tend to be. This could be exemplified by Parvarchaeum microbes, which optimally grow at the lowest temperatures but have the largest genomes among the Parvarchaeales, and the significant negative correlation of Jingweiarchaeales MAGs between OGTs and genome sizes (see Fig. S15 at https://doi.org/10.6084/m9.figshare.22126736.v3). Particularly, Parvarchaeum is the only genus that evolved nearly complete pathways for glycolysis, the TCA cycle, and the ETC for energy production. In addition, several genes, including *sqr*, *sat*, and *cysH*, were detected in Parvarchaeum, suggesting that sulfur metabolism may play vital roles during energy generation and substrate cycling. Taken together, we conjecture that oxygen may have driven the genomic diversification within Parvarchaeales.

Finally, notable genomic differences were observed between the two orders. The capacity for fermentation was more prevalent in deep-branching lineages, which were also identified to be anaerobes. Considering that the emergence of life may predate the occurrence of the great oxidation event (GOE), anaerobic respiration is likely to be ancestral across different lineages of life (74). Within DPANN archaea, it has also been shown that the ETC pathway may be absent in the common ancestor of DPANN (75). Another previous study on Parvarchaeales indicated that the TCA cycle was potentially acquired via HGT (20). The detection of HGTs in genomes in this study revealed a ratio comparable to that in a previous study (12), and it also revealed that HGT plays a vital role in the dissemination of genes associated with oxygen tolerance, which is also supported by phylogenetic inferences (Tables S1 and S8; see also Fig. S16 to S18 at https://doi.org/10.6084/m9.figshare.22126736.v3). Different origins of *sodA* genes were observed in different members. The *sodA* genes in Acidifodinimicrobium and Parvarchaeum seem to be acquired from “*Ca*. Thermoplasmatota,” while Rehaiarchaeum likely evolved this gene from *Sulfolobales* (see Fig. S16 at https://doi.org/10.6084/m9.figshare.22126736.v3). The phylogeny of the *ahpC* gene suggested that *Marsarchaeales* might be the potential donor (see Fig. S17 at https://doi.org/10.6084/m9.figshare.22126736.v3). Likewise, many of the thioredoxin-dependent peroxiredoxins encoded by *bcp* in Jingweiarchaeales and Parvarchaeales are also likely to be transferred horizontally from *Marsarchaeales*. Few *bcp* genes seem to have bacterial origins (see Fig. S18 at https://doi.org/10.6084/m9.figshare.22126736.v3). All of these results demonstrate that anaerobic respiration might be ancestral, at least for jingweiarchaeal and parvarchaeal lineages.

10.1128/msystems.01252-22.10 TABLE S8 List of detected horizontal gene transfers (HGTs) and functions of the associated genes. Download Table S8, XLSX file, 0.3 MB. Copyright © 2023 Rao et al. 2023 Rao et al. https://creativecommons.org/licenses/by/4.0/ This content is distributed under the terms of the Creative Commons Attribution 4.0 International license.

### Conclusion.

In this study, we have revealed a new order, Jingweiarchaeales, containing two previously undescribed genera, Jingweiarchaeum and Haiyanarchaeum, that is closely related to Parvarchaeales. With the analyses of metabolic potentials, we revealed that functional differentiation occurred between Jingweiarchaeales and Parvarchaeales. Microbes from Jingweiarchaeales rely mostly on a substrate salvage and fermentative lifestyle to harness energy, while the functional capacities of Parvarchaeales are more versatile, with the possession of nearly complete glycolysis, TCA cycle, and ETC pathways, etc. Comparative genomics demonstrated that Jingweiarchaeales represent a thermophilic lineage, and Haiyanarchaeum in particular may represent a hyperthermophilic genus. Further analyses revealed that the thermal adaptation of these lineages might rely on genomic features such as the usage of specific amino acids, genome streamlining, and hyperthermophile featured genes such as *rgy*. Adaptation to acidic environments by Jingweiarchaeum and Parvarchaeales requires the acquisition of genes associated with either proton export, the stabilization of inner cell environments via an impermeable cell membrane, or the decarboxylation of organic acids. Specifically, the niche expansion of Parvarchaeales was also driven by oxygen stress. By predictions of OGTs and pIs and the ancestral reconstruction of ancestral features, we demonstrated that both orders have a hot origin, and genomic expansion driven by HGT has led to adaptation to acidic-mesothermal environments. This study provides insight into the adaption and evolution of DPANN archaea in various extreme environments.

## MATERIALS AND METHODS

### Sampling, DNA extraction, and sequencing.

All 28 hot spring sediment samples were collected from Tengchong, Yunnan Province, China, in January 2016; May and September 2017; March and August 2018; January, July, and December 2019; and August 2020. Samples DRTY-1, DRTY-3, DRTY-4, DRTY-5, DRTY-6, DRTY-7, DRTY-8, DRTY-9, and DRTY-18 were collected from DiReTiYanQu (DRTY), which is an artificial concrete hot spring landscape-experiencing area. Sample ZMQR was collected from the right side of Zimei Spring, a boiling hot spring. Sample ZZQ was collected near a boiling hot spring called Zhenzhu Spring. These samples were all collected from Rehai National Park, Tengchong, Yunnan, China (24.95N, 98.44E). JZ-1 and JZ-2 were sampled from 2 concrete cubic hot spring water pools called JinZe Hot Spring Resort (25.44N, 98.46E). Detailed information about all 28 samples, including sampling dates, pHs, temperatures, and other geochemical properties, is available in Table S2 in the supplemental material. We collected the top 1 cm of sediment from each site with a sterile iron spoon and transferred these samples to a 50-mL centrifuge tube. All sediment samples were then stored in liquid nitrogen before transport to the lab. Samples were stored at −20°C in the lab until DNA extraction.

Community DNA was extracted using the PowerSoil DNA isolation kit (MoBio), from about 15 g of sediment in each sample. We used an M220 Focused-ultrasonicator instrument and a NEBNext Ultra II DNA library prep kit to build libraries with an insert size of 350 bp. The concentration of genomic DNA was measured with a Qubit fluorometer. The total genomic DNA was sequenced with an Illumina HiSeq 4000 instrument at Beijing Novogene Bioinformatics Technology Co., Ltd. (Beijing, China). Each sample was used to generate 30 Gbp of raw sequencing data.

### Metagenomic assembly and genome binning.

Raw sequencing data were preprocessed according to a previously described workflow (76) to remove replicated reads and trim low-quality bases. All quality reads of each data set were *de novo* assembled by using SPAdes v3.9.0 (77), with the parameters: -k 21,33,55,77,99,127 –meta. In each assembly, scaffolds with a length of <2,500 bp were removed. BBMap v38.92 (http://sourceforge.net/projects/bbmap/) was used to calculate the coverage information by mapping clean reads to corresponding assembled scaffolds without cross-mapping using the parameters k = 15 minid = 0.97 build = 1. Three software programs, CONCOCT (v1.1.0) (78), Maxbin2 (v2.2.7) (79), and MetaBAT (version 2.12.1) (80), were used for automated binning to generate candidate bins for each sample. The best bins were selected by using DAS Tool (v1.1.3), based on the single-copy gene (SCG) scoring rank of the predicted completeness and contamination of each bin (81). To further improve the quality of the genomes, all generated bins were mapped with quality reads of the corresponding metagenomes with BBMap. Next, the mapped reads of each bin were reassembled with SPAdes using the following parameters: -k 21,33,55,77,99,127 –careful. CheckM v1.1.3 (82) was used to estimate the contamination and strain heterogeneity of the reassembled bins, while the metric completeness was calculated using modified CheckM, which is mentioned below. Moreover, dRep (v3.2.2) (37) was used to dereplicate all 61 associated genomes in this study at a 99% ANI (strain level), and 21 genomes were selected for phylogenetic reconstruction (see Fig. S1b at https://doi.org/10.6084/m9.figshare.22126736.v3). In addition, the relative abundance of each lineage (see Fig. S2 at https://doi.org/10.6084/m9.figshare.22126736.v3) was calculated based on the coverage of the 21 representative genomes across metagenomic samples with the occurrences of lineages in this study.

### Genome quality assessment.

The genome qualities of the MAGs were assessed using three methods: (i) 48 previously described SCGs (17); (ii) CheckM2, which includes models for the genome quality assessment of DPANN superphylum archaea (34); and (iii) CheckM1, with the exclusion of markers that were missing in all genomes of each genus. For CheckM1, 9 of the 149 universal markers of archaea were missing in the genomes of all of the lineages in this study, including PF01849.13, PF01912.13, PF01922.12, PF04127.10, PF05221.12, PF06026.9, TIGR00336, TIGR00670, and TIGR01213. For each specific genus, nine markers were missing in Jingweiarchaeum (PF01849.13, PF01912.13, PF01922.12, PF04127.10, PF05221.12, PF06026.9, TIGR00336, TIGR00670, and TIGR01213), 21 were missing in Haiyanarchaeum (PF00398.15, TIGR02076, PF06418.9, PF01725.11, PF04019.7, TIGR00270, TIGR00057, PF00832.15, TIGR00549, PF01982.11, PF00958.17, PF01864.12, PF01849.13, PF01912.13, TIGR00670, PF06026.9, PF01922.12, PF04127.10, PF05221.12, TIGR00336, and TIGR01213), 29 were missing in Rehaiarchaeum (PF00900.15, PF00466.15, TIGR02338, PF04010.8, TIGR00422, TIGR00344, PF06418.9, PF01282.14, PF01725.11, PF04019.7, PF00831.18, TIGR00057, TIGR03677, TIGR00432, PF13685.1, PF00832.15, TIGR00549, PF01982.11, PF00958.17, PF01864.12, PF01849.13, PF01912.13, TIGR00670, PF06026.9, PF01922.12, PF04127.10, PF05221.12, TIGR00336, and TIGR01213), 24 were missing in Acidifodinimicrobium (PF02005.11, PF08071.7, TIGR02076, PF01725.11, PF04019.7, PF00831.18, TIGR00057, TIGR03677, TIGR00432, PF13685.1, PF00832.15, TIGR00549, PF01982.11, PF00958.17, PF01864.12, PF01849.13, PF01912.13, TIGR00670, PF06026.9, PF01922.12, PF04127.10, PF05221.12, TIGR00336, and TIGR01213), and 17 were missing in Parvarchaeum (TIGR03677, TIGR00432, PF13685.1, PF00832.15, TIGR00549, PF01982.11, PF00958.17, PF01864.12, PF01849.13, PF01912.13, TIGR00670, PF06026.9, PF01922.12, PF04127.10, PF05221.12, TIGR00336, and TIGR01213).

### Functional annotation of genomes.

An annotation pipeline was conducted to analyze genomes comparatively on a local server. Briefly speaking, putative protein-coding sequences (CDSs) of 28 MAGs were identified using Prodigal v2.6.3 under the -p single model. Functional annotations were implemented by comparing the predicted CDSs against the KEGG database (83) with the KofamKOALA (84) and arCOG (85) databases (see Tables S1 and S2 in the supplemental material). DIAMOND v2.0.11.149 (86) was used to implement the comparisons against the databases mentioned above, with a cutoff of 1e−5. Carbohydrate-active enzymes (CAZys) (49) were annotated using the local version of dbcan2 (dbscan) with the following parameters: –hmm_eval 1e−5 –dia_eval 1e−5 (87). Annotation results were kept if CAZys were identified by at least two of the three tools (HMMER, DIAMOND, and eCAMI [88]). rRNA coding regions were determined by using barrnap v0.9 (https://github.com/tseemann/barrnap). To identify tRNAs, tRNAscan-SE v2.0.9 (89) was used for all MAGs. Conserved domains of certain proteins were identified using the CD-Search tool (https://www.ncbi.nlm.nih.gov/Structure/bwrpsb/bwrpsb.cgi) of the NCBI Conserved Domain Database.

### Phylogenetic and phylogenomic analyses.

A total of 286 reference genomes, including 33 parvarchaeon-associated genomes, 218 genomes from other DPANN lineages, and 35 genomes from TACK archaea, were carefully selected from public databases for phylogenomic analyses (Table S7). As reported in a previous study, 53 concatenated proteins were selected to reconstruct the phylogeny (32) (updated in GTDB r207 [https://gtdb.ecogenomic.org/stats/r207]). These marker sequences were identified by AMPHORA2 (90) and aligned using MUSCLE v3.8.31 (91) by iteration 100 times. Poorly aligned regions were trimmed via TrimAl v1.4.rev22 (92), setting the parameters -gt 0.95 -cons 50. The phylogenetic tree was constructed using IQ-TREE v1.6.11 (93) with the parameter -alrt 1000 -nt AUTO.

10.1128/msystems.01252-22.9 TABLE S7 List of reconstructed ancestral traits, including OGT (IVYWREL), OGT (Tome), pI bias, and average pI. Download Table S7, XLSX file, 0.01 MB. Copyright © 2023 Rao et al. 2023 Rao et al. https://creativecommons.org/licenses/by/4.0/ This content is distributed under the terms of the Creative Commons Attribution 4.0 International license.

Reference RuBisCO sequences were selected from a previous study (41). These sequences were aligned with MAFFT v6.864b (94). Poorly aligned regions were removed using TrimAl v1.4.rev22. The unrooted phylogeny was generated using IQ-TREE with the following parameters: -alrt 1000 -bb 1000 -m JTT.

The phylogenetic analyses of specific proteins of interest, including reverse gyrase, superoxide dismutase, AhpC/TCA family peroxiredoxin, and peroxiredoxin, were conducted using the same procedure. First, protein sequences were extracted from the associated proteomes of the genomes in this study. Next, they were searched against the NCBI-nr database, and the top 100 hits for each sequence were kept for further analysis. The CD-hit package (95) was applied to reduce sequence redundancy. Finally, sequence alignment, the removal of poorly aligned regions, and phylogenetic tree construction were conducted using the same methods as the ones used for RuBisCO.

### Comparative genomics analyses.

The amino acid identity (AAI) between genome pairs was determined by calculating the mean identities of orthologs, which were extracted from all reciprocal best BLAST hits (rBBHs) (E value of <1e−10). Heatmaps of AAI and amino acid usage were generated by the pheatmap package (v1.0.12) (96) in R. Rate smoothing of the phylogenetic tree on the heatmaps was conducted with the makeChronosCalib() and chronos() functions of the ape package (v5.6-2) (97). The optimal growth temperatures (OGTs) were calculated using the OGT_prediction program (27), Tome (26), and the amino acids frequencies of 7 amino acids (IVYWREL) (23). Principal-coordinate analysis (PCoA) was conducted based on the copy numbers of arCOGs/KOs detected in each MAG with the vegan package (v2.6-2) in R (98). Genomic feature differences were tested by a Wilcoxon signed-rank test using the wilcox.test() function in the stat package (v4.1.1) (99) in R. Data visualizations were conducted with the ggplot2 package (v3.3.6) (100).

The calculation of the isoelectric point (pI) based on the amino acid sequences of each genome was conducted using the standalone version of the isoelectric point calculator (IPC) program (http://isoelectric.org/) (68) trained with the data from the proteome-pI database (http://isoelectricpointdb.org/) (69). The proteome of each genome was further divided into “acidic” and “nonacidic” proteins according to their pI values. The breakpoint of the two categories was determined by the “trough” of the pI probability density function of the associated proteome of the genome, which was further used to calculate the pI bias of the associated genome.

### Detection of putative horizontal gene transfers.

Putative HGTs were identified for each genome using HGTector v2 (101). The database for HGT detection was previously customized to optimize the search quality for DPANN archaea (12). The “search” step was conducted with default parameters. For the “analyze” step, taxonomy identifier 1462422 (“*Candidatus* Parvarchaeota”) was selected as “selfTax”, and taxonomy identifier 1783276 (DPANN superphylum) was treated as “closeTax”.

### Ancestral-trait reconstruction.

To reconstruct ancestral states for traits of interest, including the OGT and pI, we applied the fastAnc() function in the phytools package (v1.2-0) in R (102). This function is based on Felsenstein’s contrast algorithm (103), which could be used for maximum likelihood estimation (MLE) of the ancestral state. The reconstructed ancestral states were mapped with the contMap() function in the same package.

### Data availability.

The metagenome-assembled genomes described in this study have been deposited in the NCBI under BioProject accession number PRJNA544494, BioSample accession numbers SAMN31079384 to SAMN31079411. Supplemental materials, including Fig. S1 to S18, Tables S1 to S8, and supplemental results and data of this study, have been placed in Figshare (https://doi.org/10.6084/m9.figshare.22126736.v3).

10.1128/msystems.01252-22.1 TEXT S1 Annotations of all MAGs in the KofamKOALA database. Download Text S1, TXT file, 5.2 MB. Copyright © 2023 Rao et al. 2023 Rao et al. https://creativecommons.org/licenses/by/4.0/ This content is distributed under the terms of the Creative Commons Attribution 4.0 International license.

10.1128/msystems.01252-22.2 TEXT S2 Annotations of all MAGs in the arCOG database. Download Text S2, TXT file, 3.1 MB. Copyright © 2023 Rao et al. 2023 Rao et al. https://creativecommons.org/licenses/by/4.0/ This content is distributed under the terms of the Creative Commons Attribution 4.0 International license.
